# *CIAPIN1* and *ABCA13* are markers of poor survival in metastatic ovarian serous carcinoma

**DOI:** 10.1186/s12943-015-0317-1

**Published:** 2015-02-18

**Authors:** Dag Andre Nymoen, Arild Holth, Thea E Hetland Falkenthal, Claes G Tropé, Ben Davidson

**Affiliations:** Department of Pathology, Oslo University Hospital, Norwegian Radium Hospital, Montebello, N-0310 Oslo, Norway; Department of Oncology, Oslo University Hospital, Norwegian Radium Hospital, N-0310 Oslo, Norway; Department of Gynecologic Oncology, Oslo University Hospital, Norwegian Radium Hospital, N-0310 Oslo, Norway; University of Oslo, Faculty of Medicine, Institute of Clinical Medicine, N-0316 Oslo, Norway

**Keywords:** Ovarian serous carcinoma, Effusions, Chemotherapy response, Survival

## Abstract

**Background:**

The objective of this study was to investigate the expression and clinical role of 14 genes previously shown to be associated with chemotherapy response and/or progression-free survival in a smaller series of ovarian serous carcinoma effusions.

**Methods:**

Advanced-stage serous ovarian carcinoma effusions (n = 150) were analyzed for mRNA expression of *AKR1C1*, *ABCA4*, *ABCA13*, *ABCB10*, *BIRC6*, *CASP9*, *CIAPIN1*, *FAS*, *MGMT*, *MUTYH*, *POLH*, *SRC*, *TBRKB* and *XPA* using quantitative real-time PCR. mRNA expression was studied for association with clinicopathologic parameters, including chemotherapy response and survival.

**Results:**

*ABCA4* mRNA expression was significantly related to better (complete) chemotherapy response at diagnosis in the entire cohort (p = 0.018), whereas higher *POLH* mRNA levels were significantly related to better chemoresponse at diagnosis in analysis to 58 patients with pre-chemotherapy effusions treated with standard chemotherapy (carboplatin + paclitaxel; p = 0.023). In univariate survival analysis for patients with pre-chemotherapy effusions (n = 77), *CIAPIN1* mRNA expression was significantly related to shorter overall (p = 0.007) and progression-free (p = 0.038) survival, whereas *ABCA13* mRNA expression was significantly related to shorter OS (p = 0.024). Higher *CIAPIN1* mRNA expression was an independent marker of poor overall survival in Cox multivariate analysis (p = 0.044).

**Conclusions:**

Our data identify *ABCA4* and *POLH* as markers of better chemotherapy response in metastatic serous carcinoma. *CIAPIN1* and *ABCA13* may be novel markers of poor outcome in pre-chemotherapy serous carcinoma effusions.

## Introduction

Ovarian cancer, consisting predominantly of ovarian carcinoma (OC), is the most lethal gynecologic malignancy, constituting the 5th and 4th most common cause for cancer-related death in women in the U.S. and Norway, respectively [1,2]. Standard therapy consists of surgical cytoreduction followed by adjuvant chemotherapy using carboplatin and paclitaxel. While the majority of patients will respond to chemotherapy, the majority of tumors will recur within a median period of 18 months and become chemoresistant, resulting in 5-year survival of 30% for advanced-stage disease [3]. Mechanisms by which cancer cells acquire resistance to platinum agents include reduced cellular uptake, increased efflux, increased DNA repair and hypermethylation of *MLH1*, part of the mismatch repair (MMR) system. Resistance to paclitaxel occurs through overexpression of multi-drug resistance protein 1 (MDR1), tubulin mutations and chromosomal instability [4]. Our current understanding of the molecular events that mediate chemoresistance in recurrent and metastatic OC is limited by the paucity of studies focusing on extra-ovarian disease.

OC progresses predominantly within the abdominal cavity and to a lesser degree by metastasis to the pleural space, frequently with the formation of malignant effusions. The serosal cavities are additionally a frequent site of disease recurrence [5]. We previously analyzed 32 serous OC effusions for the expression of 381 genes which have been reported to be associated with chemoresistance in OC using TaqMan arrays, and identified genes which were significantly associated with overall survival (OS), progression-free survival (PFS) and/or chemotherapy response [6]. In addition to its limited size, the latter study analyzed together primary diagnosis pre-chemotherapy effusions and effusions obtained post-chemotherapy, predominantly at disease recurrence. The objective of the present study was to analyze the expression of 14 genes found to be associated with chemoresponse and/or PFS in a larger cohort, consisting of 150 patients with serous OC effusions.

## Materials and methods

### Patients and material

Fresh non-fixed malignant peritoneal (n = 125) and pleural (n = 25; total = 150) effusions were obtained from 150 patients with FIGO stage III or IV serous ovarian, peritoneal or tubal carcinoma treated at the Department of Gynecologic Oncology at the Norwegian Radium Hospital in the period 1998–2008. Due to their closely-linked histogenesis and phenotype, all are referred to as OC henceforth. Informed consent was obtained according to national guidelines. The study was approved by the Regional Committee for Medical Research Ethics in Norway. Clinicopathologic data are detailed in Table 1.

**Table 1:** **Clinicopathologic parameters of the study cohort (150 patients)**

**Parameter**		**Number of patients**
**Age**		35-87 years (mean = 62)
**Histological grade**	Low	3
	High	120
	NA^*a*^	27
**FIGO stage**	III	83
	IV	67
**Residual disease**	≤1 cm	59
	>1 cm	63
	NA^*b*^	28
**CA 125 at diagnosis** ^***c***^		11-43800 years (mean = 3375)
**Chemoresponse after primary treatment** ^***d***^	CR	78
	PR	32
	SD	9
	PD	21
	NE	10

^*a*^NA = non available, including effusions from inoperable patients where biopsy was too small for grading or patients operated on in other hospitals, for which the primary tumor could not be accessed for assessment of grade.

^*b*^NA = non available, including 4 patients who only received chemotherapy and 1 patient who received no therapy.

^*c*^CA 125 levels available for 104 patients.

^*d*^CR = complete response, PR = partial response, SD = stable disease, PD = progressive disease, NE = disease response after chemotherapy could not be evaluated because of adverse effects, normalized CA-125 after primary surgery or missing CA-125 information and no residual tumor.

Eighty-six patients had primary surgery, 56 had secondary debulking and 4 patients only received chemotherapy. One patient received no therapy, and 3 patients referred at disease recurrence had no data available regarding therapy at diagnosis. Seventy-seven samples were obtained prior to chemotherapy administration, and 69 were obtained after chemotherapy, at interval debulking surgery or at recurrent disease. Chemotherapy status was unknown in 4 cases. The majority of patients (n = 138) received platinum-based therapy, of whom 101 received platinum + paclitaxel, 27 received single carboplatin and 10 received platinum combination. Of the remaining 12 patients, 10 received other chemotherapy and 1 received no therapy, whereas no information was available for 1 patient.

Effusions were submitted for routine diagnostic purposes and were processed immediately after tapping. Cell blocks were prepared using the Thrombin clot method. Diagnoses were established using morphology and immunohistochemistry (IHC). Effusion specimens were centrifuged immediately after tapping, and cell pellets were frozen at −70°C in equal amounts of RPMI 1640 medium (GIBCO-Invitrogen, Carlsbad CA) containing 50% fetal calf serum (PAA Laboratories GmbH, Pasching, Austria) and 20% dimethylsulfoxide (Merck KGaA, Darmstadt, Germany). Smears and H&E-stained cell block sections were reviewed by a surgical pathologist experienced in cytopathology (BD). The tumor cell population was >50% in all specimens.

### Quantitative real-time PCR (qRT-PCR)

Effusions were centrifuged to obtain a cell pellet from which RNA was extracted using QIAsymphony (Qiagen, Hilden Germany). Details regarding reverse transcription, primer and probe design procedure and software, and efficiency testing were recently described [7]. The qRT-PCR reaction was run using the Perfecta qPCR ToughMix (Quanta Biosciences, Gaithersburg MD) and quantified on the Roche LightCycler 480 (Roche, Basel, Switzerland). Samples were analyzed in triplicate and average copy number was used for statistical analysis. Primer and probe sequences are detailed in Table 2.

**Table 2:** **Primers and probes**

**Gene**	**Sequence**
*ABCA4*	F-ACTTCTTCAAGCTCTTCCGTGTGC
NM_000350.2	R-TCAGATAATATTCCTCCCCAAGATCTCAG
EXON 6-7	P-CCACACTCCTAGACAGCCGTTCTCAAGGTATC
*ABCA13*	F-CTGTGGAAGAATTGGCTCTGCA
NM_152701.3	R-CAGAATTACAAACAGGATACAAGGCCAG
EXON 1-2	P-CTCAGGAACCCGGTCCTTTTCCTTGCTGAATTC
*BIRC6*	F-CACGTCCAGAACTCGGAGTG
NM_016252.3	R-AGGTAAATGTCTCCCGTCTGTTAGC
EXON 4-5	P-AGGCCGTTCTGTAGACAGATCACTGATGTATAGTG
*CASP9*	F-CAGACCAGTGGACATTGGTTCTG
NM_001229.3	R-CTCAGGATGTAAGCCAAATCTGCAT
EKSON 3-4	P-TGGTGATGTCGGTGCTCTTGAGAGTTTGAG
*CIAPIN1*	F-CACCAAGAAGTCTTCTCCTTCAGTG
NM_020313.2	R-GCTGAGAGGGTCCACAGCT
EXON 5-6	P-ACCTGCTGTGGACCCTGCTGCTG
*MGMT*	F-CATCCCGTTTTCCAGCAAGAG
NM_002412.3	R-GGTAAGAAATCACTTCTCCGAATTTCA
EXON 3-4	P-TTCACCAGACAGGTGTTATGGAAGCTGCTGAAG
*TPRKB*	F-GGGAGCTCTTCCGAGACG
NM_016058.2	R-GTCCAGCTGATGTGTTAACTGCAT
EXON 1-2	P-TGGGGGCCGGATGTAGAATCCTGCTTA
*XPA*	F-CACAATGGGGTGATATGAAACTCTACT
NM_000380.3	R-CCTTTGCTTCTTCTAATGCTTCTTGACT
EXON 4-5	P-AAGTTACAGATTGTGAAGAGGTCTCTTGAAGTTTGGG
*POLH*	F-TATGTCCAGATCTTCTACTGGCACAAGT
NM_006502.2	R-AAAACGAGACATTATCTCCATCACTTCA
EXON 3-4	P-CGTGGGAAAGCTAACCTCACCAAGTACCGG
*FAS*	F-GATTGCTCAACAACCATGCTG
NM_000043.4	R-GGGCATTAACACTTTTGGACGAT
EXON 1-2	P-TGGACCCTCCTACCTCTGGTTCTTACGTCTGTTGCTAG
*SRC*	F-CCCCTGCCTTCTACCAGGAC
NM_005417.3	R-CGGCGGGCTCCAGGCT
EXON 3-4	P-TGGGTAGCAACAAGAGCAAGCCCAAGGATG
*AKR1C1*	F-CATCAGACAGAACGTGCAGGTG
NM_001353.5	R-CAAAAATATCAAGGGTCAAATATCGC
EXON 7-8	P-CCAGTTGACTTCAGAGGAGATGAAAGCCATAGATG
*MUTYH*	F-TCCACCGCCATGAAAAAGGT
NM_001048171.1	R-GACCTTTTGGAACCCATACAGGTC
EXON 14-15	P-TCCGTGTGTATCAGGGCCAACAGCCA
*ABCB10*	F-ACTCTCTTCCTTCCTAATGTATGCTTTCTG
NM_012089.2	R-CACCCAGTCCTTTCATCAGCT
EXON 6-4	P-TGGAATAAGCATTGGAGGTCTGAGCTCTTTCTACTC

F = Forward primer; R = Reverse primer; P = Probe. Probes are labeled with 5′Fam and 3′non fluorescent quencher.

The beta-glucuronidase (*GUS*), TATA box binding protein (*TBP*) and mitochondrial ribosomal protein L19 (*MRLP19*) genes were used as reference genes following previous testing [7] applying established guidelines [8-10]. Primer and probe sequences were previously detailed [7].

### IHC

Formalin-fixed paraffin-embedded sections from 109 of the 150 effusions analyzed using qRT-PCR were analyzed for Ciapin1 protein expression using the Dako EnVision™ Flex + System (K8012; Dako, Glostrup, Denmark). Deparaffinization and unmasking of epitopes were carried out in a PT-Link (Dako) using an EnVision™ Flex target retrieval solution at a low pH (Tris/EDTA pH 6). Sections were incubated with a 0.3% hydrogen peroxide (H_2_O_2_) solution for 5 min to block endogeneous tissue peroxidase activity. Sections were incubated with a rabbit polyclonal Ciapin1 antibody (NBP1-89097, Novus Biologicals, Littleton, CO; 1:100 dilution), and then treated with EnVision™ Flex + mouse linker (15 min) and EnVision™ Flex/HRP enzyme (30 min). Sections were stained for 10 min with 3’3-diaminobenzidine tetrahydrochloride (DAB), counterstained with hematoxylin, dehydrated and mounted in Richard-Allan Scientific Cyto seal XYL (Thermo Fisher Scientific, Waltham, MA). Positive and negative controls consisted of bladder carcinoma and sections stained with rabbit serum, respectively.

#### Interpretation

Staining was considered positive when localized to the cytoplasm or nucleus. Staining extent was scored on a scale of 0–4, corresponding to staining of 0%, 1-5%, 6-25%, 26-75% and 76-100% of cells. No specimen contained less than 100 tumor cells. Slides were scored by a surgical pathologist experienced in effusion cytology (BD).

### Statistical analysis

Statistical analysis was performed applying the SPSS-PC package (Version 18, Chicago IL). Probability of <0.05 was considered statistically significant. Analysis of the association between mRNA levels of the 14 studied genes, as well as Ciapin1 protein expression, and clinicopathologic parameters was performed using the Mann–Whitney *U* test. For this analysis, as well as for survival analysis, clinicopathologic parameters were grouped as follows: Age: ≤60 vs. >60 years; effusion site: peritoneal vs. pleural; FIGO stage: III vs. IV; chemotherapy status: pre- vs. post-chemotherapy specimens; Residual disease (RD): ≤1 cm vs. >1 cm; chemotherapy response: complete vs. partial response/stable disease/progression; primary (intrinsic) chemoresistance: PFS ≤6 months vs. >6 months. Histological grade was not used for statistical analyses due to the small number of low-grade tumors. The paired-sample *T*-test was used to analyze the association between mRNA expression levels of the studied genes and CA 125 levels.

PFS and OS were calculated from the date of the last chemotherapy treatment/diagnosis to the date of recurrence/death or last follow-up, respectively. Univariate survival analyses of PFS and OS were executed using the Kaplan-Meier method and log-rank test. Platinum resistance was defined as PFS ≤ 6 months according to guidelines published by the Gynecologic Oncology Group (GOG) [11] and progressive disease or recurrence was evaluated by the RECIST criteria [12]. Multivariate analysis was performed using the Cox Regression Model (Enter function).

## Results

### Chemoresistance-associated genes are significantly related to clinicopathologic parameters

Analysis of mRNA levels for the 14 studied genes in the 150 effusions identified several significant associations with anatomic site, patient age and residual disease volume (Table 3). No significant associations were found with chemotherapy status (pre- vs. post-chemotherapy specimens) or with FIGO stage (p > 0.05).

**Table 3:** **Significant associations between gene expression and clinicopathologic parameters in the entire study cohort (150 patients)**

**Parameter**	**Gene**	**Overexpression**	**P-value**
**Anatomic site**	*ABCA13*	Peritoneum	p = 0.03
	*FAS*	Pleura	p = 0.041
**Age**	*ABCB10*	Age >60 years	p = 0.006
	*BIRC6*	Age >60 years	p = 0.004
	*ABCA13*	Age >60 years	p = 0.011
	*CASP9*	Age >60 years	p = 0.049
**Residual disease**	*XPA*	≤1 cm	p = 0.046
	*MGMT*	≤1 cm	p = 0.04
	*TBRKB*	≤1 cm	p = 0.002
	*ABCB10*	≤1 cm	p = 0.011
**CA 125 levels at diagnosis**	*ABCA4*	Positive association	p = 0.011
	*ABCB10*	Negative association	p = 0.01

Our previous research has demonstrated that pre- and post-chemotherapy effusions differ considerably with respect to expression of cancer-associated molecules and their clinical relevance [5]. Despite the absence of significant differences in expression of the 14 studied genes among pre- and post-chemotherapy effusions, we therefore analyzed them separately.

Significant associations for pre-chemotherapy cases are shown in Table 4. In analysis of post-chemotherapy effusions, only age was significantly associated with gene expression, with associations identified between older age and lower *ABCA4* (p = 0.02) and higher *BIRC6* (p = 0.03), *ABCA13* (p = 0.008) and *FAS* (p = 0.018) levels.

**Table 4:** **Significant associations between gene expression in pre-chemotherapy effusions and clinicopathologic parameters (77 patients)**

**Parameter**	**Gene**	**Overexpression**	**P-value**
**Anatomic site**	*FAS*	Pleura	p = 0.034
**Age**	*ABCB10*	Age >60 years	p = 0.005
**FIGO stage**	*MUYTH*	III	p = 0.03
	*ABCA4*	III	p = 0.011
**Residual disease**	*MGMT*	≤1 cm	p = 0.007
	*TBRKB*	≤1 cm	p = 0.003
	*ABCB10*	≤1 cm	p = 0.014
**CA 125 levels at diagnosis**	*ABCA4*	Positive association	p = 0.003
	*ABCB10*	Negative association	p = 0.042

**Figure 1:**
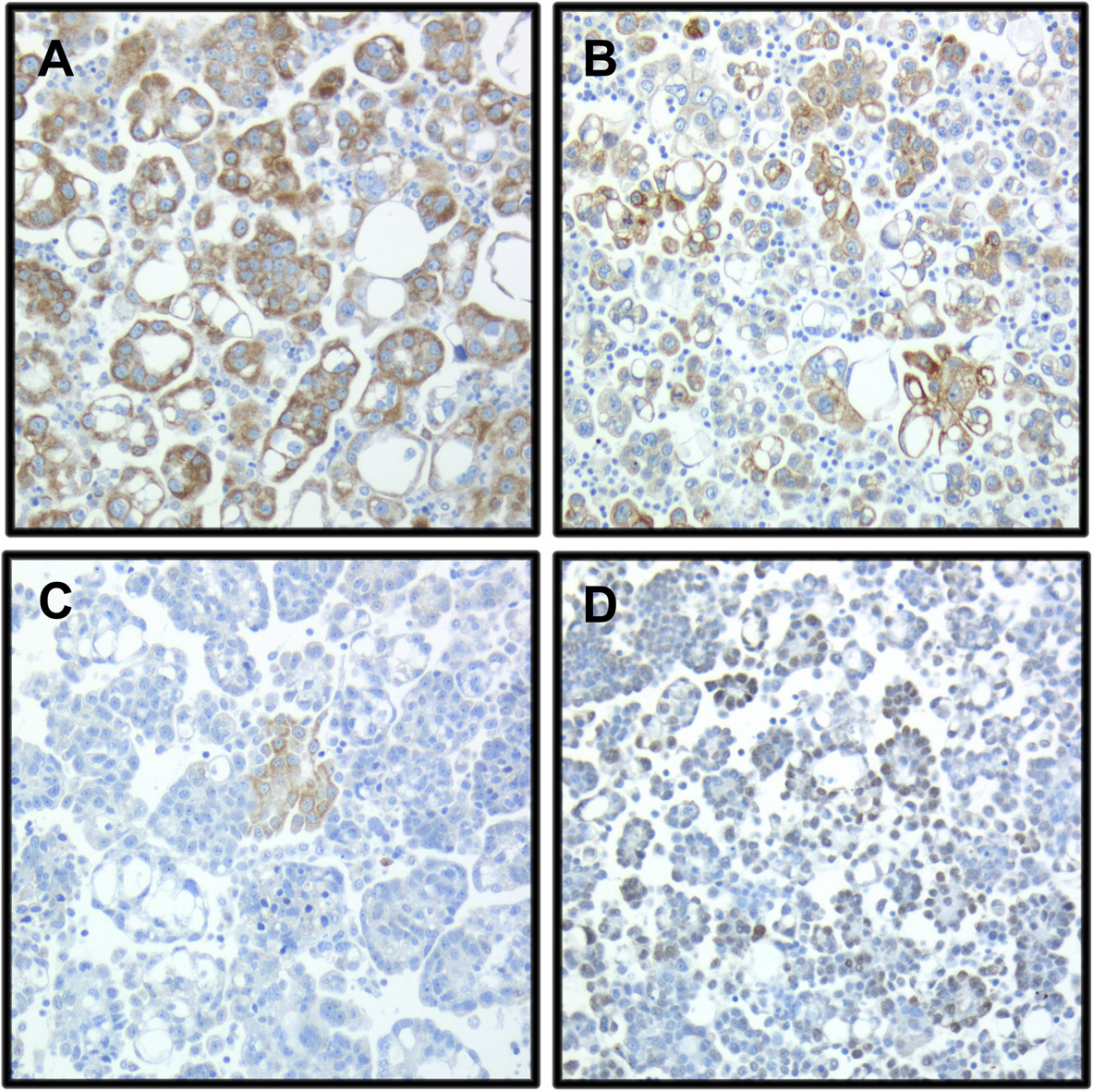
**Ciapin1 protein expression by immunohistochemistry. (A-B)** Diffuse Ciapin1 expression (>75% of tumor cells) in the cytoplasm of OC cells in 2 effusions; **(C)** Focal expression (<5%) in another specimen; **(D)** Nuclear localization of Ciapin1, an unusual finding in this series.

Ciapin1 protein was expressed in the cytoplasm of OC cells in 92/109 effusions, of which 28 had score = 1, 25 score = 2, 24 score = 3 and 15 score = 4 (Figure 1). Nuclear localization was observed in <10 cases and was not scored (Figure 1). Expression in reactive mesothelial cells was observed in 2 cases, while staining was tumor-specific in the remaining effusions.

### *ABCA4* and *POLH* mRNA expression is related to complete chemotherapy response at diagnosis

In analysis of the 144 patients which had data regarding chemotherapy response at diagnosis, higher *ABCA4* mRNA expression was significantly related to better (complete) chemoresponse at diagnosis (p = 0.018). In evaluation of the relationship between gene expression and primary chemoresistance in the entire cohort, no significant associations were found (p > 0.05). However, trends for association between high *ABCA13* (p = 0.059) and low *FAS* (p = 0.053) mRNA levels with primary chemoresistance were observed.

In analysis of the 73 patients with pre-chemotherapy effusions which had data regarding chemotherapy response at diagnosis, higher *CIAPIN1* mRNA expression was associated with a trend for worse (non-complete) chemoresponse at diagnosis (p = 0.052). A trend was additionally found between low *FAS* mRNA levels and primary chemoresistance (p = 0.056).

Limiting the analysis to 58 patients with pre-chemotherapy effusions treated with standard chemotherapy (carboplatin + paclitaxel) identified *POLH* mRNA as marker better chemoresponse at diagnosis (p = 0.023). Ciapin1 protein expression was unrelated to chemotherapy response.

CA 125 level at diagnosis, available for 59 patients with pre-chemotherapy effusions, was not significantly related to chemoresponse at diagnosis (p = 0.207). HE4, which is currently measured in all OC patients at our hospital, was not measured in the period the effusions studied in this series were collected (1998–2008), and therefore cannot be assessed for performance in this study.

None of the studied genes was related to chemoreponse at diagnosis or to primary chemoresistance in the group of patients with post-chemotherapy effusions (p > 0.05).

### *CIAPIN1* and *ABCA13* are prognostic markers in pre-chemotherapy serous OC effusions

The follow-up period for the 150 patients ranged from 1 to 156 months (mean = 33 months, median = 24 months). PFS, available for 149 patients, ranged from 0 to 116 months (mean = 9 months, median = 5 months). At the last follow-up, 1 patient was alive with no evidence of disease, 6 were alive with disease and 142 were dead of disease. One patient died of unrelated causes.

**Figure 2:**
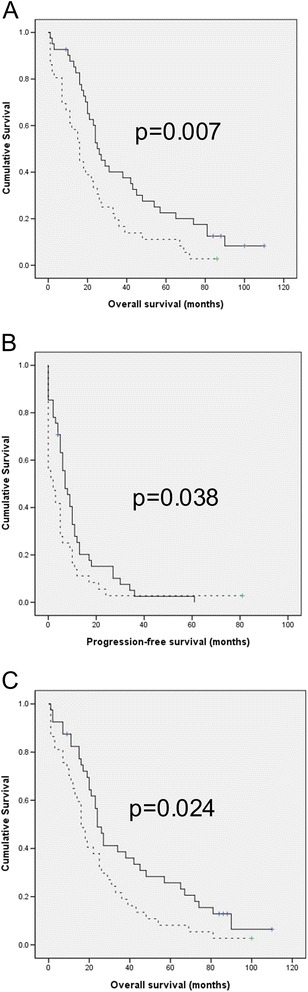
***CIAPIN1*** **and** ***ABCA13*** **mRNA expression is associated with shorter survival in serous ovarian carcinoma. A**: Kaplan-Meier survival curve showing the association between *CIAPIN1* mRNA expression in effusions and overall survival (OS) for patients with pre-chemotherapy primary diagnosis effusions (n = 77). Patients with effusions with higher-than-median expression (n = 36, dashed line) had a mean OS of 22 months vs. 40 months for patients whose effusions showed lower-than-median expression (n = 41, solid line; p = 0.007). **B**: Kaplan-Meier survival curve showing the association between *CIAPIN1* mRNA expression in effusions and progression-free survival (PFS) for patients with pre-chemotherapy primary diagnosis effusions (n = 77). Patients with effusions with higher-than-median expression (n = 36, dashed line) had a mean PFS of 7 months vs. 11 months for patients whose effusions showed lower-than-median expression (n = 41, solid line; p = 0.038). **C**: Kaplan-Meier survival curve showing the association between *ABCA13* mRNA expression in effusions and OS for patients with pre-chemotherapy primary diagnosis effusions (n = 77). Patients with effusions with higher-than-median expression (n = 36, dashed line) had a mean OS of 24 months vs. 39 months for patients whose effusions showed lower-than-median expression (n = 41, solid line; p = 0.024).

In survival analysis for all patients, none of the studied genes was significantly related to OS or PFS. The same findings were observed for the patient group with post-chemotherapy effusions. However, in univariate survival analysis for patients with pre-chemotherapy effusions (n = 77), *CIAPIN1* mRNA expression was significantly related to shorter OS (p = 0.007; Figure 2-A) and PFS (p = 0.038; Figure 2-B). In addition, *ABCA13* mRNA expression was significantly related to shorter OS (p = 0.024; Figure 2-C), though not with PFS (p = 0.192). *ABCA4* and *POLH* expression was not significantly related to survival, and the same was true for Ciapin1 protein expression.

Among the clinical parameters, older age was significantly related to shorter OS (p = 0.044) and was associated with a trend for poor PFS (p = 0.055). RD volume >1 cm was significantly related to poor PFS (p = 0.031), with non-significant association with OS (p = 0.196). CA 125 level at diagnosis in patients with pre-chemotherapy effusions were unrelated to PFS (p = 0.185) or to OS (p = 0.413) in this patient group.

The parameters entered into Cox analysis of OS and PFS were all those with p < 0.2, i.e. *CIAPIN1* and *ABCA13* mRNA expression, RD volume and age for both endpoints. In Cox multivariate analysis of OS, higher *CIAPIN1* mRNA expression was the only independent marker of poor outcome (p = 0.044). In Cox multivariate analysis of PFS, larger RD was the sole independent marker (p = 0.041).

In univariate survival analysis for patients with pre-chemotherapy effusions who received combination chemotherapy with carboplatin and paclitaxel (n = 58), *ABCA13* mRNA expression (p = 0.006) and older age (p = 0.026) were significantly related to shorter OS. Non-significant associations with shorter OS were observed for *CIAPIN1* mRNA expression (p = 0.146) and RD volume (p = 0.141). In Cox multivariate analysis, none of these markers was an independent prognosticator.

## Discussion

Intrinsic and acquired drug resistance remains a major therapeutic obstacle in OC [3], and our understanding of the cellular mediators of this phenomenon in metastatic OC cells is limited by the paucity of large series of such specimens. In the present study we assessed the predictive and prognostic role of 14 genes identified as potentially clinically relevant in an earlier study [6]. Our data only partially confirm the initial report. Inconsistent results from different mRNA expression studies are not infrequent and different methodology is the major cause of these discrepancies [13]. In our case the initial study used a Taqman array assay and *18S* for normalization vs. our in-house assay in which primers were designed by our group and the average of 3 reference genes previously shown to be optimal for OC analysis was used for normalization. The number of samples also differed considerably, being 32 in the previous study vs. 150 in the present one. Some findings were nevertheless reproduced, as *CIAPIN1* and *ABCA13* expression had prognostic value in pre-chemotherapy patients and *ABCA4* and *POLH* were predictive for complete chemotherapy response at diagnosis. Additional correlation with clinopathologic parameters was found for anatomic site (*ABCA13* and *FAS*), patient age (*ABCB10*, *BIRC6*, *ABCA13* and *CASP9*) and residual disease volume (*XPA*, *MGMT*, *TBRKB* and *ABCB10*). Though no association was observed between gene levels and previous chemotherapy or FIGO stage in analysis of the entire cohort, FIGO stage was significantly associated with *MUTYH* and *ABCA4* expression in pre-chemotherapy disease.

Three members of the ATP binding cassette (ABC) family were assessed on the present study. These transmembrane proteins serve as efflux pumps and members like MDR1 play a major role in multidrug chemoresistance [14,15]. The role of ABCA4 in cancer is largely unknown to date, whereas ABCB10 was previously shown to be expressed in drug-resistant HT-29 colon carcinoma cells [16]. Higher *ABCA13* levels by qPCR have been associated with better response to neoadjuvant chemotherapy in breast cancer [17] and with longer disease-free survival following chemotherapy in colorectal cancer [18], and this gene was previously reported to be underexpressed in renal cell carcinoma compared to normal tissue [19]. Our data associate *ABCA4* with better chemotherapy response at diagnosis. However, the marginal association between higher *ABCA13* levels and primary chemoresistance and the significant association with poor OS suggest that this molecule may have a different role in serous OC.

The present study identified *CIAPIN1* as an independent prognostic marker of poor outcome for patients with pre-chemotherapy effusions. Cytokine induced apoptosis inhibitor 1 (CIAPIN1) is an anti-apoptotic protein exerting its function through post-translational modifications of cell cycle proteins including p27, cyclins D1 and E and the cyclin-dependent kinases CDK2 and CDK4. It additionally cooperates with other proteins, such as thioredoxin-like protein 2 (TXNL2), to inhibit apoptosis via the mitochondrial (intrinsic) pathway. Ciapin1 additionally regulates cell differentiation, organ development and cellular stress response [20]. Nuclear expression of CIAPIN1 was reported to be associated with poor prognosis in a study of 108 primary OC [21]. In our series, this protein was predominantly localized to the cytoplasm, nuclear expression being too infrequent to be considered of clinical relevance. This emphasizes the differences between primary OC and effusion specimens, but may also reflect the fact that the study by Cai et al. included both serous and non-serous tumors, whereas our series consisted uniformly of the former.

*POLH*, encoding Polymerase η, was associated with complete response to primary chemotherapy in the patient group with pre-chemotherapy effusions treated with standard chemotherapy (carboplatin + paclitaxel). POLH, a.k.a. XPV and RAD30A, is member of the Y-family of DNA polymerases. Mutation in this protein is the basis for the Variable type of Xeroderma Pigmentosum, a genetic disease with impaired DNA repair associated with skin cancer [22]. Polymerase η was further reported to mediate resistance to cisplatin by extending primers after DNA cross-linking induced by the drug [23]. Our data do not support this role and rather constitute the first report attributing a potential beneficial clinical role to this molecule in OC.

The remaining genes analyzed in this study were not informative for chemotherapy response or survival. Protein expression of FAS, activator of the extrinsic apoptosis pathway, by flow cytometry in OC effusions was previously reported to be a marker for aggressive clinical behavior by our group [24], but its mRNA was significantly related only to anatomic localization (pleura vs. peritoneum) in the present study. Similarly, protein expression of the DNA repair gene XPA was previously found to be associated with better prognosis by our group [25], whereas *XPA* mRNA expression was unrelated to survival in the present study. These discrepancies likely reflect different methodology, as well as the fact that protein and mRNA expression do not fully overlap in many cases.

The inhibitor of apoptosis (IAP) family consists of 8 proteins which are key regulators of apoptosis. IAP members are overexpressed in many cancers and are under considerable attention as therapeutic targets [26,27]. Protein expression of the IAP member Apollon (a.k.a. Baculoviral IAP Repeat Containing 6; BIRC6) in primary OC was recently reported to be related to poor prognosis [28], an association which was not reproduced in our analysis of *BIRC6* mRNA levels in effusions. Similarly, SRC, modulator of response to paclitaxel and a potential molecular target in OC [29], was not informative of therapy response or survival in our series.

TPRKB, a.k.a. TP53RK, binds and activates p53 by phosphorylation. The gene is located in the 20q13.12-13 region which is amplified in OC and was reported to be associated with primary chemoresistance [30]. TP53RK silencing activates caspases 3 and 7 and sensitizes OC cells to paclitaxel [31].

MUTYH is a DNA glycosylase that repairs oxidative damage by replacing an incorrectly placed A with C, thereby preventing mutations. However, it appears to have additional roles, primarily as pro- and anti-apoptotic marker, and is localized to both the mitochondria and the nucleus. MUTYH is implicated in cancer, inflammation and Parkinson disease. Mutations in *MUTYH* predispose individuals to colorectal cancer [32], as well as increased risk of developing OC [33].

O-6-methylguanine-DNA methyltransferase (MGMT), an enzyme regulated primarily through promoter methylation, repairs DNA damage caused by alkylating lesions and thus rescues tumor cells from apoptosis [34]. Higher MGMT activity was reported to be associated with cisplatin resistance in OC [35].

AKR1C1 is family member of the Aldo-keto reductases, and have been reported to enhance progesterone metabolism in ovarian endometriosis [36].

A relevant question concerning the results of the present study would be how other biomarkers perform in this cohort with respect to predicting chemotherapy response or as prognostic factors. We recently published a retrospective analysis of 143 serous OC effusions in which we assessed the clinical role of 41 previously studied proteins. In this series, several proteins that were significantly related to chemotherapy response and/or survival (the latter in pre-chemotherapy effusions) were identified, including survivin, fatty acid synthase (FAS), p21-activated kinase-1 (PAK1), osteopontin, peroxisome proliferator-activated receptor-γ (PPARγ), S100A4, Bcl-XL, high-mobility group protein A2 (HMGA2), NAC-1, signal transducer and activator of transcription 5B (STAT5B) and heat shock protein 90 (HSP90) [37]. Analysis of the clinical role of these markers in the pre-chemotherapy specimens analyzed in the present study identified only nuclear survivin expression as significantly related to chemotherapy response (favorable; p = 0.017) or OS (favorable; p = 0.013). It should nevertheless be commented that the number of overlapping specimens was 47 or less for these markers. We are currently performing a similar multivariate analysis for biomarkers studied at the mRNA level, which may complement the current study in identifying clinically relevant targets in OC effusions.

In conclusion, analysis of 14 chemoresistance-associated genes in a large series of serous OC effusions identified *CIAPIN1*, *ABCA4*, *ABCA13* and *POLH* as potential markers of survival or chemotherapy response in this setting, findings which merit in our opinion further research. Multiple associations were additionally identified between the studied genes and various clinicopathologic parameters. While some of the latter may represent errors of multiple testing, the stronger associations (p < 0.005) may represent true findings. Sorting the clinically relevant from the non-relevant molecular markers in recurrent and/or metastatic OC may aid in selection of therapeutic targets in this cancer.
